# Saw His Own Heart

**Published:** 1896-06

**Authors:** 


### ARTICLE VIII.

### SAW HIS OWN HEART.

(Chicago Letter in "Philadelphia Times.")

Charles W. Whitney, one of the policemen who was injured ten years ago in the Chicago Haymarket riot has had a curious experience. His chest was torn away by the explosion of a bomb, and as he lay in the hospital bed he saw, with the aid of a hand mirror his own throbbing heart. What is still more marvelous, he recovered, and is to-day doing a man's work, and commands $10 a year more than an able-bodied policeman traveling a beat.

May 4, 1886, was a bloody night in Chicago history.

One hundred and seventy stalwart men marched from the Desplaines Street Station. They had been corralled there for several days anticipating a crisis, and at 10:30 o'clock the order"fall in line" was given. The country thrilled with the horror that followed; their ineffectual efforts to disperse the mob of frenzied Anarchists, the throwing of a bomb that burst between Lieutenants Stanton and Bowler's companies left over sixty officers lying in a heap on Haymarket Square. One man died within an hour and two others were hopelessly crippled by having their legs blown off, other men were cruelly maimed, fourteen of whom are now dead.

But of all the men Whitney stood the smallest possible chance of life. His breast was horribly mutilated, and bits of exploded bomb were embedded perilously near the vital organs. He was taken to the hospital, and, after the necessary probing, the wound was sewed up.

Just before taking the stitches Dr. J. B. Murphy inquired:

"My brave boy, do you want to see what no man ever saw before?"

"Yes; what is it?" asked the officer feebly.

"Your own heart!"

He had handed his patient a mirror, and Whitney saw the greatest miracle ever given to human eye. He now affirms that the statement of novelists is misleading; that the heart is not a movable organ, for it neither leaps up into the throat nor descends into the boots. In fact, it does not indulge in any of the acrobatic feats generally ascribed to it by imaginative writers. But he frankly acknowledges that he is incapable of expressing his sensation as he watched the oscillation of his heart.

Within a month he was able to return home and was supposed to be on the road to recovery. Later he suffered a relapse, and after weeks of unparalleled suffering, Dr. Murphy decided to resort to the most extreme operations known to scientific surgery. In the history of surgical procedure in this country it was the third time it has ever been undertaken, the former cases having proved fatal. A careful examination indicated that a piece of the bomb casing penetrated the breast bone, passing about midway between the second and third ribs and was located about one-eighth of an inch from the pericardium. Like a magnet coquetting with a piece of metal, every throb of the heart brought the piece of shell nearer, until the piercing of the organ was inevitable.

The operation was set for Friday, November 6, 1886. Relatives and friends of the injured man protested, and this coming to his ears, he said.

"I am going to make a test case of this, and see if there is anything wrong with Friday."

The eventful morning came. Up to this time Whitney had been exceptionally hopeful and cheery.

"I never realized that I could die until just as I was getting under the influence of the ether. There were fourteen doctors in the room and among them was an old family physician who was with my mother when I was born. Before the anesthetic took effect, one of the physicians began shaving the hair on my chest. A sudden horror seized me and in my dazed condition I imagined it was a fight for life. I, who had been so weak that I could scarcely raise my hand to my head, suddenly struck the one who was doing the tonsorial act a terrible blow. I kicked another doctor in the stomach and another in the chest. I fought like a mad creature, and it took eleven men to hold me while I was getting under the influence of the drug."

The old wound was completely healed, but the flesh was laid open and Dr. Murphy proceeded to bore a hole through the officer's breast bone, very much as a skillful carpenter uses an augur on a hard wood stick. The instrument used was a trephine, suitable for making a hole five-eighths of an inch in diameter. It took eighty minutes to cut through the breast bone which at this point is nearly an inch thick.

The delicate operation was a marvelous success, for at the foot of this tiny tunnel lay the piece of bomb, which was easily plucked out with a pair of tweezers. It was cone-shaped, and composed of almost equal parts of copper, zinc and lead, and was cosily ensconced in the outer fatty coating of the heart.

It was over a year before it healed, and during that time he wore a rubber drainage tube. But it was several years before he was able to do any manual labor. Meanwhile he watched the Anarchists' trial, kept in touch with his comrades at the station, and was detailed for light service. Then for three years he drove a patrol wagon, and afterwards served as a messenger on the force. For a couple of years he was officer at the Goodrich School, and two years ago he was appointed at the Carter Harrison Public Bath, where his star awes unruly bathers, large and small. He draws a regular salary for his services, and as long as he lives he will have a pension. The law provides for this, but the citizens of Chicago will see that there is an assured living for every man hurt in the Haymarket riot.

Whitney is a handsome man, with large gray eyes and brown moustache. Suffering has given his face the characteristics of a scholar rather than a man of muscle. His conversation with physicians has given him a scientific knowledge of anatomy, which is perhaps not so remarkable, considering he is the only man in the world who has seen his own heart.

---

